# Energy Metabolism in the Inner Retina in Health and Glaucoma

**DOI:** 10.3390/ijms22073689

**Published:** 2021-04-01

**Authors:** Hanhan Liu, Verena Prokosch

**Affiliations:** Department of Ophthalmology, Faculty of Medicine and University Hospital Cologne, University of Cologne, 50937 Cologne, Germany; hanhan.liu@uk-koeln.de

**Keywords:** energy metabolism, glaucoma, mitochondrial function, retinal ganglion cell, retinal blood flow

## Abstract

Glaucoma, the leading cause of irreversible blindness, is a heterogeneous group of diseases characterized by progressive loss of retinal ganglion cells (RGCs) and their axons and leads to visual loss and blindness. Risk factors for the onset and progression of glaucoma include systemic and ocular factors such as older age, lower ocular perfusion pressure, and intraocular pressure (IOP). Early signs of RGC damage comprise impairment of axonal transport, downregulation of specific genes and metabolic changes. The brain is often cited to be the highest energy-demanding tissue of the human body. The retina is estimated to have equally high demands. RGCs are particularly active in metabolism and vulnerable to energy insufficiency. Understanding the energy metabolism of the inner retina, especially of the RGCs, is pivotal for understanding glaucoma’s pathophysiology. Here we review the key contributors to the high energy demands in the retina and the distinguishing features of energy metabolism of the inner retina. The major features of glaucoma include progressive cell death of retinal ganglions and optic nerve damage. Therefore, this review focuses on the energetic budget of the retinal ganglion cells, optic nerve and the relevant cells that surround them.

## 1. Introduction

Glaucoma, one of the leading causes of irreversible blindness, affects more than 70 million people worldwide. It is a heterogeneous group of diseases characterized by progressive retinal ganglion cell loss (RGCs). This leads to structural and functional damage to the optic nerve, visual loss, and blindness [1]. Glaucoma is a comprehensive term for a heterogeneous disease comprising multiple etiologies [2].

Risk factors for the onset and progression of primary open-angle glaucoma (POAG), the most common form of glaucoma, include systemic and ocular factors, such as age, ocular perfusion pressure, and intraocular pressure (IOP).

Elevated IOP is one of the main risk factors across different types of glaucoma [3]. Reducing IOP is the mainstay of treatment in order to slow down neurodegeneration. However, many patients with glaucoma continue to progress even when IOP is controlled [4,5,6].

Considerable effort has been dedicated in previous studies to elucidate the pathology of glaucoma and to identify the cascade of structural and functional alterations in RGCs that eventually lead to their apoptosis [7]. Early signs of damage comprise impairment of axonal transport, downregulation of specific genes and metabolic changes [8,9,10,11].

The brain is one of the highest energy-demanding tissues of the human body. It consumes 20% of the energy supply while comprising only about 2% of the body weight [12,13]. The energy consumption of the retina is in a similar range to the brain [14,15,16,17,18]. The function of retinal neurons is often impacted when metabolites and oxygen supply are not meeting the high demands of the retinal neurons.

Retinal ganglion cells (RGC) possess an exceedingly active metabolism and are particularly vulnerable to energy insufficiency [19]. Therefore, understanding the retina’s energy metabolism, especially of the RGCs, is pivotal for understanding the pathophysiology of glaucoma.

Blood flow, oxygen supply, glucose utilization, and mitochondrial function are all important factors in considering energy metabolism and are all tightly interlinked with neuronal function and survival in the retina. Increased IOP and age, the other two main risk factors of glaucoma, are both directly correlated with increased ROS and impaired mitochondrial function [20,21]. Numerous studies suggest that the pathogenesis of glaucoma is potentially related to mitochondrial dysfunction. The prevalence of POAG increases with age, while the optimal function of mitochondria decreases with age, and the RGCs critically rely on mitochondrial for their function and survival [22,23,24,25]. The RGC appears highly susceptible to primary or secondary mitochondrial dysfunction. Many neurodegenerative diseases with proven mitochondrial defects show specific loss of this neuronal population, such as in Leber’s hereditary optic neuropathy (LHON), a classical mitochondrial disease characterized by selective RGC loss [26,27].

Here we review the key contributors to the high energy demands in the retina and the distinguishing features of energy metabolism of retinal neurons. The major features of glaucoma include progressive cell death of retinal ganglions and optic nerve damage [28]. The focus of this review is, therefore, on the energetic budget of the retinal ganglion cells, optic nerve and the relevant cells that surround them.

## 2. High-Energy Demands in the Retina

The retina is not a structurally or functionally unified organ, and it is the most complex of the ocular tissues. The neuronal component of the retina is composed of six types of neurons: photoreceptors (rods and cones), bipolar cells, horizontal cells, amacrine cells and retinal ganglion cells (RGCs) [29]. The retinal neurons receive the visual stimulus and convert the light energy into electrical signals, which undergo a tremendous amount of processing within retinal layers prior to transmission by the optic nerve to the visual cortex in the brain. The various retinal neurons have specific tasks with varying energy demands; energy metabolism in the retina is complicated. Neuronal activity is tightly coupled with energy metabolism at both cellular and molecular levels. When metabolites and oxygen supply are not meeting their high demands, their activities may be vitiated [19].

### 2.1. Types of Energy Metabolism in Retina

The metabolic and energy needs of the retina have been assumed to be met by glucose, as the retina is part of the CNS, and the brain relies almost exclusively on glucose [30,31]. Energy is generated from glucose by two interrelated metabolic pathways: glycolysis in the cytoplasm and oxidative phosphorylation (OXPHOS) in the mitochondria [32]. Retinal metabolic activity can be evaluated by measuring retinal metabolic rates of oxygen and glucose [33]. Energy supply in the inner retina is supported by both aerobic and anaerobic pathways, but the anaerobic pathways are much less pronounced than in the outer part [34]. Indicators of anaerobic glycolysis, such as the lactate formation, the H^+^ levels and the lactate dehydrogenase (LDH) activity, are low in the inner retina [16,35,36].

There is also a substantial portion of the energy produced through oxidation by the retina (around 65%) was not derived from glucose [37]. Photoreceptors can oxidize lipid to produce ATP, accounting for the energy gap noted by Cohen et al. [38,39].

#### 2.1.1. Glucose Supply and Metabolism in the Retina

Retinal neurons rely more on a ready supply of glucose than glial-derived lactate for their energy production [40]. Blood-derived glucose must pass through outer and inner BRB to reach retinal neurons. Glucose transporters (*GLUT*) present on both BRB facilitates glucose passage to the retina [41,42]. *GLUT1* is the major glucose transporter present in the retina. In humans, expression of *GLUT1* is reported in both the retinal capillary endothelial cells and the retinal pigment epithelium [42].

Glycolysis is an anaerobic process, which breaks down six-carbon monosaccharides through a series of enzyme-catalyzed reactions that yield two molecules of three-carbon compound pyruvates. During glycolysis, glucose in the retina can be converted into pyruvate and then transported into the mitochondria and fully oxidized, or in anaerobic conditions, pyruvate is converted into lactate, yielding substantially less ATP. The majority (~80%) of the glucose supplied to the avascular outer retina by the choroid is converted into lactate via glycolysis, in comparison to the vascularized inner retina, where only 20% of glucose supplied by the retinal circulation is converted to lactate [34,37].

High lactate production usually occurs when oxygen is limited. German physiologist Otto Heinrich Warburg noticed that cancerous tissue tended to divert glycolytic metabolites towards biosynthesis rather than towards oxidative phosphorylation, therefore, produce ATP via incomplete glucose oxidation despite the presence of oxygen [43,44]. This is called aerobic glycolysis, also known as the Warburg effect [44].

It is not fully understood why normal tissue relies on aerobic glycolysis over oxidative phosphorylation of glucose. A possible explanation for the Warburg effect in the retina is that due to the mitochondria’s localization to the inner segment, the outer segment is forced to rely on aerobic glycolysis [45]. This speculation is supported by the distribution of lactate dehydrogenase (LDH) involved in glycolysis in the outer segment of the retina [46]. Warburg effect is indeed a manifestation of both high energy demands and relatively reduced oxygen consumption, considering that lactate is the end product of glycolysis [47].

The cell types that carry out aerobic glycolysis in the normal adult retina are yet to be determined. The photoreceptors are assumed to be the primary site of aerobic glycolysis in the retina, as they possess enzymes for aerobic glycolysis and produce a certain quantity of lactate under physiological conditions [48,49]. Müller cells contain very few mitochondria and must rely mainly on anaerobic glycolysis for energy; glucose is metabolized primarily to lactate in retinal Müller glial cells [50,51,52].

Nevertheless, the purpose of aerobic glycolysis in the retina, its cell origin and relevance to photoreceptor and their regulation require to be further studied.

#### 2.1.2. Oxygen Consumption and Oxidative Phosphorylation in the Retina

The energetic advantage of oxidative phosphorylation (OXPHOS) in mitochondria far outweighs that of glycolysis aerobic, as per molecule of glucose yields only 2 molecules of net ATP via the glycolytic pathway and 36 molecules of ATP in the mitochondria.

The retina is one of the most oxidative tissues in the body. Oxygen consumption reflects mitochondrial activity and its production of ATP. The inner segments of photoreceptors have a very high oxidative metabolism, but since the rest of the photoreceptor has no mitochondria, the outer half of the retina does not have an extraordinarily high metabolic rate [48]. Oxygen tension is highest near the choroid and rapidly decreases moving towards the photoreceptors and increases again after passing the photoreceptor mitochondrial layer towards the inner retina [53].

Cytochrome c oxidase and the terminal complex (complex IV) of the electron transport chain in the inner mitochondrial membrane is a major regulation site for oxidative phosphorylation [54,55]. In general, the distribution pattern of cytochrome c oxidase in the retina reflects regions of energy production and usually matches the energy demand [56]. An increase in cytochrome c oxidase expression typically reflects mitochondrial reparative activity, while a decrease in cytochrome c oxidase is regarded as a hallmark of neurodegeneration [57]. Cytochrome c oxidase is nonhomogeneously distributed in the retina and optic nerve [58]. Within the retina, high enzyme activity levels were found localized within the retinal ganglion cells and nerve fiber layer, the outer plexiform layer, inner segments of photoreceptors, and the retinal pigment epithelium [59]. In the optic nerve, the unmyelinated prelaminar and laminar regions were rich in both cytochrome c oxidase and succinate dehydrogenase. Myelination of fibers as they exited the lamina cribrosa was associated with an abrupt reduction in enzyme activity [59].

### 2.2. Neuronal Energy Demands to Sustain Retinal Function

The specific mediators that link neuronal metabolism with retinal angiogenesis in the developing eye and retinal disease remain largely unknown. However, it is becoming more evident that the metabolic needs of the neural retina profoundly influence the vascular network that supplies oxygen and nutrients [55].

Some of the energy-consuming functions of neurons comprise the synthesis of proteins, the loading, release, recycling, and turnover of neurotransmitter molecules, active transport of macromolecules and organelles along microtubules between the cell bodies and their dendrites and especially axons [60,61,62,63]. However, these processes account for only a minor portion of energy consumption.

The most energy-consuming function of neurons by far appears to be the active transport of ions against their concentration and electrical gradients [64,65,66]. Active ion transport is supported mainly by Na+K+ATPase [67], which utilizes 1 ATP for every 3 Na^+^ pumped out in exchange for 2 K^+^ brought into the cell [68]. It serves mainly to repolarize the plasma membrane after depolarization, to reestablish the transmembrane ionic gradient for reactivation [69].

As the distribution pattern of cytochrome c oxidase reflects the regions of energy production in the retina, the differential distribution of Na+K+ATPase denotes the reductions in energy demand.

The Na+K+ATPase is densely localized in the inner segments of photoreceptor cells, the outer plexiform layer, and the nerve fiber layer, whereas the inner plexiform layer and ganglion cells have moderate levels of this enzyme [70].

The biggest ATP demand in the retina comes from photoreceptors and the retinal pigment epithelium (RPE) [71]. Photoreceptors are the first-order neurons of the visual pathway, converting light into electrical signals [48]. Na+ K+ ATPase in the inner segments consumes the most of the energy to pump out excess Na+ entering via cGMP-gated channels in the outer segments in the dark, thereby maintaining the dark current [72]. Other than maintaining the dark current, photoreceptor cells are actively engaged in energy-dependent light transduction. Light induces the isomerization of the chromophore 11–cis-retinal to an all-trans-retinal, which is then reduced to an all-trans-retinol [73]. The majority of retinal oxidative phosphorylation (OXPHOS) occurs in photoreceptors [74], which is accounts for more than 60% of the oxygen consumption of the retina [14]. Consistent with their high energy demand, photoreceptors retain over 60% of retinal mitochondria in their inner segments [39], as well as the highest electron transport chain enzyme cytochrome C oxidase activity [75]. The RPE is a close interaction partner of the photoreceptors; in a function critical to phototransduction, the RPE uptake all-trans-retinol from the photoreceptors and convert it to 11-cis-retinal and recycle back to photoreceptor cells [73]. The RPE cell is also responsible for maintaining the volume and chemical composition of the subretinal space that would otherwise change with the light and dark cycle [76]. The electrogenic sodium pumps actively transport ions and assists in the transport of metabolites and fluid across this cell layer [77].

Cytochrome c oxidase, as well as Na+K+ATPase, are also densely localized in the retinal nerve fiber layer, where the ganglion cell axons are unmyelinated, which denotes a certain level of depolarizing and repolarizing activities [70,75]. The high energy demands of the unmyelinated axons render them vulnerable to energy failure. Retinal ganglion cells and their axons are easily affected in conditions where the energy metabolism is compromised, or blood flow is restricted, such as in Leber’s hereditary optic neuropathy (LHON) and diabetic retinopathy [78,79]. Particularly in glaucoma, where RGCs are selected to die, elevated IOP and other risk factors potentially impair mitochondrial function and retinal vascular function [80,81]. The energy metabolism of RGCs in health and glaucoma is to be discussed below in detail.

### 2.3. Blood Supply of the Retina

Neuronal energy demands are met by a tightly coupled and adaptive vascular network that supplies oxygen and nutrients like glucose, which both contribute to the final yield of ATP in mitochondria [53]. A decrease in blood flow below a critical level can cause a 90% drop in ATP within 5 minutes in the brain and leads to cell death [82].

Retinal vessels are precisely regulated to optimize blood supply to meet the large metabolic needs of the retina without interfering with the visual pathway [83].

The mature retina in humans and other primates is supplied by two vascular networks. The outer half of the retina, including the outer plexiform and outer nuclear layers, the photoreceptors, and the retinal pigment epithelium is supplied by choriocapillaris; the inner half of the retina is supplied by the inner retinal vasculature originates from branches of the central retinal artery (CRA), which in turn is a branch of the ophthalmic artery. The vasculature in the inner retina is further layered in plexuses that form a retinal neurovascular unit [84].

The outer retina’s oxygen needs are met predominantly from the choroidal circulation, and the oxygen needs of the inner retina are met from the retinal circulation [85].

Retinal circulation is characterized by a low-level flow and high levels of oxygen extraction [86]. It has no autonomic innervation and is autoregulated [87,88]. The relatively large arterio-venous oxygen difference in the retinal circulation suggests that significant oxygen consumption in the inner retina [89]. The inner retina is generally believed to be more susceptible than the outer retina under impaired perfusion. Ocular perfusion pressure is equal to the difference between the mean arterial blood pressure (BP) and intraocular pressure, which is an important determinant of ocular blood flow [90]. High IOP-induced ocular ischemia is a frequently utilized glaucoma animal model to mimic “glaucoma attack” in acute angle-closure glaucoma. An acute attack of angle-closure glaucoma describes an acute IOP elevation due to acutely closing the angle between the iris and cornea [91]. Not only the retina and the optic nerve but also other ocular tissues, such as lens, iris and iris sphincter, can be affected by the irreversible ischemic damages from the attack, resulting in irregular pupil, iris atrophy and glaukomflecken.

The IOP is elevated above ocular perfusion pressure, which leads to global ischemia with obstruction of both the retinal and uveal circulation, as evidenced by whitening of the iris and the fundus. The high IOP ischemia model characteristically injures the inner retina to a much greater extent than the outer retina [92].

The potential role of impaired blood flow to the optic nerve as a cause of glaucoma has been discussed since the 19th century. A plethora of evidence has accumulated since then; the vast majority of published studies dealing with blood flow report reduced ocular perfusion in glaucoma patients compared with normal subjects. The reduction of blood flow and its velocities in the retina, choroid and ONH in glaucoma patients has been demonstrated using different detection methods [93,94,95]. Generalized narrowing of the retinal vessels is characteristic of advanced glaucomatous optic nerve damage, but blood flow reduction appears to be especially pronounced in the peripapillary area [96,97].

The major cause of this reduction is rather vascular dysregulation [86]. Autoregulation can be defined as the capability of an organ to regulate its blood supply in accordance with its metabolic demands [98]. Within the eye, autoregulation is defined as local vascular constriction or dilation, causing vascular resistance to reciprocally increase or decrease, thereby maintaining a constant nutrient supply in response to perfusion pressure changes [99]. The retinal circulation is also regulated by metabolic factors, and this could be called metabolic regulation or metabolic autoregulation. Extracellular lactate leads to contraction or relaxation of the vessel wall, depending on the metabolic needs of the tissue [100]. Retinal vessel tone can be actively regulated by the ionic or molecular factors released by the vascular endothelium or surrounding neural tissue. These factors can be relaxing or contracting to the vessel tone, such as NO is a relaxing factor, NO activity contributes to ocular autoregulation and can protect the endothelium and nerve fiber layer against pathologic stressors implicated in glaucoma and ischemia [101]. Opposing the vasodilation properties of NO is endothelin-1 (*ET-1*) and angiotensin II, which are vascular constricting factors. Altered NO activity and *ET-1* expression are documented in glaucoma patients in different studies [102,103,104,105,106].

The capacity of autoregulation may become less potent, or may completely fail, in glaucoma leading to the tissue being under-perfused [107]. Evidence suggests that impaired vascular autoregulation renders the optic nerve head susceptible to decreases in ocular perfusion pressure, increases in IOP, and/or increased local metabolic demands [2].

#### 2.3.1. Blood Retina Barrier

Retinal neurons are the most sensitive and critical cells in the eye analogs to neurons in the brain. Other than tightly regulated hemodynamics and delivery of oxygen and metabolic substrates, intact blood-retinal barriers (BRB) are also essential requirements for the maintenance of optimal retinal structure and function [83]. The two most frequent and relevant retinal diseases, diabetic retinopathy and age-related macular degeneration are directly associated with alterations of the BRB [108].

The blood-retinal barrier restricts nonspecific transport between the neural retina and the circulating blood, therefore, maintains a stable microenvironment for the neuronal cells [109,110]. It is formed by an inner and outer component. Although the retinal and choroidal vessels are all derived from the ophthalmic artery, which is originated from the internal carotid, the dual blood supply has distinctive morphological and functional differences [111]. At the level of the capillary endothelium: the central retinal artery (CRA)-derived capillaries have tight junctions like the brain, which forms the inner blood-retinal barrier, while the choriocapillaris has a fenestrated and polarized endothelium [83,112].

The inner BRB is similar to the blood–brain barrier; a functional neurovascular structure comprises the complex tight junctions of retinal capillary endothelial cells, pericytes and astrocyte foot processes [113,114].

The inner BRB efficiently supplies nutrients to the retina and removes endobiotic and xenobiotics from the retina to maintain a constant milieu in the neural retina [110]. Just like brain endothelial cells, endothelial cells in the retina also contain more mitochondria comparing with those in other parts of the body.

Recent studies have shown that mitochondria in endothelial cells have a crucial role in maintaining the blood–brain barrier and BRB [115]. Inhibition of mitochondria in cerebrovascular endothelial cells disrupts BBB integrity and increases BBB permeability in vitro and in vivo [115]. Mitochondrial dysfunction is increasingly recognized as an accomplice in vascular diseases [115]. Endothelial metabolic compromise contributes to vascular dysfunction in glaucoma [116]. Besides maintaining the integrity of BRB, endothelial cells play a major role in the local regulation of blood flow [117,118,119].

The outer BRB is formed at the retinal pigment epithelial (RPE) cell layer by the tight junctions between the RPE cells [114,120]. Unlike other epithelial cells, the apical surface of the RPE is in direct contact with neural tissue, and it is centrally involved in the daily phagocytosis of the tips of photoreceptor cells [121]. In one direction, the RPE transports electrolytes and water from the subretinal space to the choroid, and in the other direction, the RPE transports glucose and other nutrients from the blood to the photoreceptors [122]. The Na+K+ATPase, which is located apically in RPE cells, provides the energy for transporting electrolytes and water from the subretinal space to the choroid [123]. Reduction in glycolysis and mitochondrial ATP production in aged RPE is correlated with increased susceptibility to oxidative stress [124].

#### 2.3.2. Blood Supply of the Optic Nerve Head

The optic nerve head (ONH) describes the point for the ganglion cell axons exiting the globe through the lamina cribrosa. The ONH is predominately supplied by branches of the posterior ciliary artery (PCA) and recurrent choroid arteriole with the superficial nerve fiber layer supplied by branches of the central retinal artery [90,125]. Venous drainage of the ONH is through the central retinal vein. The ONH seems to be the only part of the central nervous system, which has no proper blood–brain barrier, with the capillaries lacking blood–brain barrier properties [126].

The optic nerve head remains the point at which retinal ganglion cell axons are most vulnerable to the effects of increased intraocular pressure or ischemia; the blood flow at the optic nerve head is delicately regulated to maintain the supply of oxygen and nutrients to the RGC axons [127].

Evidence accrued over the decades suggests that dysfunctional regulation of ocular blood flow contributes to glaucomatous optic neuropathy and plays a prominent role in glaucoma processes [2]. In the ONH of glaucoma patients, different kinds of blood flow defects were observed, comprising local filling defects, slow filling and increased leakage [128,129]. Increased intraocular pressure potentially restricts blood flow and can eventually lead to ischemia that would be detrimental to the optic nerve head and retinal ganglion cells [130].

### 2.4. Energy Metabolism in RGC

Retinal ganglion cells (RGC), the neurons that selectively die in glaucoma, possess an exceedingly active metabolism and are particularly vulnerable to energy insufficiency [19]. RGCs are specialized output neurons of the eye that are primed to transmit an abundant set of visual information from the retina to the brain. 90% of all sensory signals that are integrated into the brain are of visual origin [131], and everything the brain knows about the content of the visual world is built from the spiking activity of RGCs [132].

Like most neurons, RGCs are polarized into dendritic and axonal compartments that are connected to the cell body. Signal inputs are collected by the dendrites, and output is distributed from the cell body via axons [133]. Each subcellular component of the RGC is located in a different retinal layer, their somata are located along the inner margin of the retina, in the retinal ganglion cell layer (GCL), and their dendrites interlace with amacrine and bipolar cells in the inner plexiform layer (IPL). The non-myelinated RGC axons reside in the retinal nerve fiber layer, and the myelinated axons form the optic nerve. The components are also remarkably different in terms of structure and function; hence, the energy demands and distribution of each component are also distinct, as evidenced by the uneven distribution of mitochondria and ATP within the RGC, signifying the presence of intracellular energy requirements [133].

Previous studies in animal models of glaucoma also suggest that RGCs may not all be uniformly affected; diverse RGC types respond to IOP elevation at different time-scales and to varying extents [134]. Degeneration and functional loss appear to affect the neuronal processes in the dendrites and the axons well before the cell body in the retina [7,135,136]. Some studies have shown dendritic arbors of some RGCs appear more affected than others at the advanced stage of glaucoma [137,138,139], and regions of relatively unaffected cells can still be observed [140,141].

The most recent estimate of the number of distinct RGC types found in the mammalian retina is around 30, with more than half of these types definitively identified [142]. However, it is still a challenge to define the rules that govern which RGC types are most susceptible or resistant to glaucomatous injury in a comprehensive manner [143].

There also has been debate in the field as to whether RGCs with large somata and axons are more vulnerable, with definitive conclusions still in progress because of the wide diversity of RGC types [143]. While the variety of animal models in which experimental glaucoma has been studied has provided conflicting data, it also has raised recurring evidence that supports the hypothesis that the process of RGC degeneration may be compartmentalized at the subcellular level whereby independent degenerative pathways occur in the soma, axon, dendrite and their synapse [144,145,146,147].

Each subcellular component of the RGC is remarkably different in terms of structure, function and extracellular environment [133]. Rather than attempting to understand RGC as a homogeneous structure, it is more convenient to view the RGC as a series of compartments to understand the pathogenic processes involved in its degeneration in glaucoma pathogenesis [147].

**Figure 1:**
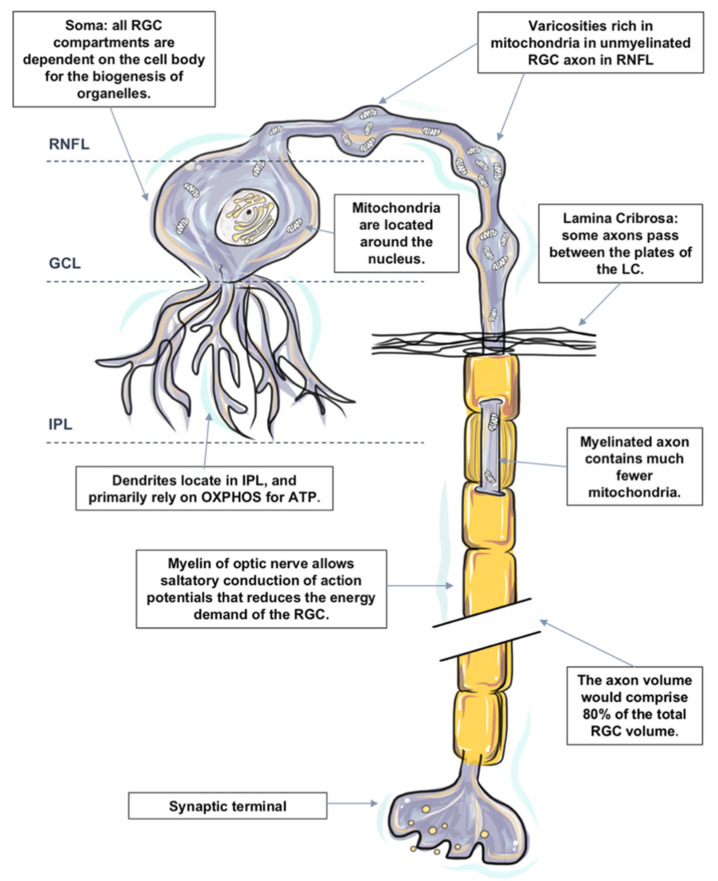
The distinguishing metabolic features of retinal ganglion cell (RGC). RGCs are polarized into dendritic and axonal compartments that are connected to the cell body. RGC bodies are located in the ganglion cell layer (GCL). All organelles, including mitochondria within an RGC, are synthesized in the cell body and then transported to targeted sites. RGC dendrites receive inputs from bipolar cells and branch in the inner plexiform layer (IPL). Despite the unique metabolic features in the IPL, RGC dendrites primarily rely on oxidative phosphorylation (OXPHOS) for ATP and are particularly susceptible to a varied range of injuries. Dendritic arbors of the RGCs within the off sublamina of IPL are among the first to undergo shrinkage and death after very brief exposure to elevated pressure. Axons of RGCs connect the eye with the brain and are under considerable metabolic stress in both health and disease states. Mitochondria are distributed asymmetrically along optic nerve axons, with regional organelle concentrations correlating closely with the local energy demands. Mitochondrial enzyme activity and immunoreactivity are higher in these unmyelinated regions. Due to the lack of salutatory conduction, the energy demands of non-myelinated axons in the RNFL are unusually high, which renders them vulnerable to disruptions that led to energy exhaustion and eventual functional failure. The initial damage following IOP elevation is speculated to occur at the axonal compartment of the cell at the lamina cribrosa of the optic nerve head. Just posterior to scleral laminar, axons are myelinated. Myelination allows salutatory conduction of action potentials that reduces the energy demand for the cell; cytochrome c oxidase level falls precipitously in the retrobulbar optic nerve.

In the following sections, we divide the RGC into the four subcellular components: (1) RGC somata located, (2) RGC dendrites and their synapses, (3) mon-myelinated axons at nerve fiber layer and at the ONH, and (4) myelinated axons in the orbit and the cranial region, which are located within the optic nerve. The distinguishing energy metabolic features and response to physiological and pathological challenges of each compartment are discussed, respectively (Figure 1).

There is a large number of studies on quantitative measurements of energy production in the retina. However, measurements specifically targeting the metabolism of RGCs remain sparse. Different techniques have been utilized to reflect the pattern of energy consumption and distribution in different subcellular components of RGCs, such as the distribution of mitochondria, the expression of cytochrome oxidase and the presence of neuroglobin.

Nonetheless, each technique also has its own technical limitations, and these are only the indices of metabolism but not necessarily linearly related to oxygen consumption. It is currently not possible to tell the exact changes in energy utilization in glaucoma. The lack of specific knowledge about energy consumption and distribution in RGC subcellular components is a limitation in our analysis of glaucoma.

#### 2.4.1. Energy Metabolism in RGC Somata

All RGC compartments are critically dependent on the cell body for the biogenesis of organelles. All organelles within an RGC are synthesized in the cell body and then transported to targeted sites. The functional activity and survival of RGC axons and dendrites are dependent upon the RGC somata [148]. Injury to the cell body has a devastating impact on the function and survival of the entire cell.

RGC bodies are located in the RGC layer. The RGC layer has a rich blood supply [27,149]. Mitochondrial biogenesis and protein occur within the cellular somata of RGC, together with high enzyme activity in the IPL and surrounding RGCs [27]. Mitochondria are generally believed to be located around the nucleus, which results in relative hypoxia [150]. Relative intracellular hypoxia surrounding the soma may play an important protective role in reducing the free radical attack injury to nucleic acids [147,151].

One RGC transmits the information from numerous photoreceptors to the brain; its cell body is significantly larger compared to other neurons in the retina. It is speculated that RGCs with larger somata and axons may be selectively vulnerable to IOP elevation [143]. When compared to normal optic nerves, glaucomatous optic nerves had greater loss of large-diameter axons, which might indicate the selective loss among RGCs [152,153]. Earlier work in non-human primates and human tissue also supported the concept that RGCs with the largest cell bodies and axons were the most susceptible to injury [154,155]. It is worth noting that this concept is highly controversial, and it did not necessarily indicate the selective vulnerability of a specific RGC type [143]. It is demonstrated in the later studies that cell soma shrinkage was likely a stage of degeneration prior to cell loss and raised the question of whether or not the previous work misidentified large vs. small RGCs because of cell shrinkage [156].

#### 2.4.2. Energy Metabolism in RGC Dendrites and their Synapses

RGC dendrites receive inputs from bipolar cells, which convey signals from photoreceptors and from amacrine cells that branch in the inner plexiform layer (IPL) [133]. The input gathered from the synaptic network is integrated and summated at the RGC dendrites before an action potential can be initiated at the axon hillock resulting in an “all or none” response [157]. The axon hillock is the site of the greatest concentration of voltage-sensitive sodium channels in neurons and is, therefore, a site of high energy consumption [158].

Comparing to other capillary networks in the retina, the networks supplying the IPL have a smaller capillary diameter, less capillary density values and a complex three-dimensional configuration [27]. Such structural adaptations are believed to maximize nutrient delivery to energy-dependent synapses while preserving the optical transparency of the inner retina. Under the hypoxic condition, more oxygen can be made available to the IPL from the choroidal circulation [159,160]. Despite the unique metabolic features in the IPL, RGC dendrites and synapses are still particularly susceptible to a varied range of injuries. One of the signs of RGC degeneration reduction in sensitivity to light and reduction of the excitatory synapses on the cell’s dendritic arbor [134,161,162]. There is conflicting evidence in regard to whether or not RGC dendrites are the first compartment to be perturbed when physiological or pathological challenges are presented to the RGCs [163,164]. In a mouse glaucoma model, dendritic arbors of certain RGC subtypes manifest significant changes in the structure after very brief exposure to elevated IOP, especially the RGCs within the off sublamina of the inner plexiform layer are among the first to undergo shrinkage and death [165]. OPA1 deficient mice (Opa1+/−), a model of autosomal dominant optic atrophy, which is also characterized by selective RGC death, display RGC dendritic atrophy and accumulation of fragmented mitochondria at dendrites prior to overt visual deficits and RGC loss [166].

#### 2.4.3. Energy Metabolism in RGC Axons

Axonal degeneration of retinal ganglion cells (RGCs) and apoptotic death of their cell bodies are observed clinically in glaucoma patients. Abnormal swelling and accumulations of mitochondria in RGC axons have been identified following prolonged IOP elevation [167]. It has been proposed that the initial damage to the axons of the RGCs occurs at the level of the lamina cribrosa [168].

Axons of RGCs connect the eye with the brain; they are under considerable metabolic stress in both health and disease states [127]. It is worth noting that the axon volume is estimated to comprise 80% of the total RGC volume based on published data [127,153,169], and the lack of saltatory conduction in the unmyelinated intraocular portion of the RGC axons both place a particular bioenergetic burden on this cellular component. While the cell soma is the principal site for energy production and protein synthesis, there is evidence that the axons supplement some of these functions as they share a similar mitochondrial profile with the somata [170].

Mitochondria are distributed asymmetrically along optic nerve axons with regional organelle concentrations correlating closely with the local energy demands; the distribution and localization of mitochondria along RGC axons are critical for normal function [171,172]. Histochemical and immunohistochemical evidence indicates that mitochondrial enzyme activity and immunoreactivity are higher in these unmyelinated regions [173,174,175].

##### Non-Myelinated Axon in the Retina and ONH

1. Nerve Fiber Layer (NFL)

The nerve fiber layer is the inner retinal layer of unmyelinated ganglion cell axons. Due to the lack of salutatory conduction, their energy demands are unusually high. There is a regional layer of a capillary network called the radial parapapillary capillary plexus, which runs in parallel with the NFL axons to supply specifically the bundles [176,177,178]. The NFL expresses a high level of cytochrome c oxidase and Na+ K+ ATPase, comparing the unmyelinated axons in the brain [70,75], as well the presence of neuroglobin and relatively dense astrocytes in the NFL [27,149,179], which indicates an abundant degree of depolarizing and repolarizing activities within. Furthermore, with the assistant of transmission electron microscopy, it is presented that the unmyelinated portion of the RGC axons in primates (including humans) display varicosities rich in mitochondria, which suggests that the local energy demands of non-myelinated axons are high [180]. The pattern of mitochondrial concentration in the unmyelinated segments resembles that of the myelinated segments, but for fibers thicker than 0.7 μm, the volume fraction is twofold greater [181].

Notwithstanding, the density of the inner retinal vasculature that is to sustain the high metabolic demands is constrained by the requirement for relative optical transparency.

The delicate metabolic balance between energy demands and energy delivery places non-myelinated axons in the NFL in a vulnerable position to a range of disruptions that led to energy exhaustion and eventual functional failure, particularly hypoxic and ischemic insults [127].

2. Axons at ONH

Nerve fibers travel from the optic nerve head through a sieve-like structure called the lamina cribrosa into the extraocular space. In vitro tracing of individual axon paths in the human, optic nerve head has shown that some axons do not take a direct course through the lamina cribrosa (LC) [182]. Some axons undergo a rather complex partial decussation at the margin of the optic nerve head, in which axons from peripheral and central retinal ganglion cells mingle to adopt the correct location within the retrobulbar optic nerve [183,184,185]. Some axons even pass between the plates of the LC instead of taking a direct path through it. It is speculated that following IOP elevation of varying duration and amplitude, the axonal compartment of the cell at the lamina cribrosa of the optic nerve head is initially affected, resulting in impaired axonal transport and initiation of the degeneration process.

3. Myelinated Axons in the Orbit and Cranial Region (Optic Nerve)

Just posterior to scleral laminar, axons are wrapped by oligodendrocytes as they enter the retrolaminar part of the optic nerve [186]. Myelination allows saltatory conduction of action potentials that reduces the energy demand for the cell; cytochrome c oxidase level falls precipitously in the retrobulbar optic nerve [75].

The axons that form the optic nerve have an average diameter of 1 mm but can range from 0.7 to 10 μm in diameter [187]. The smaller axons come from, the smaller RGCs of the central part of the retina, and the larger axons come from the RGCs of the peripheral retina [188]. The optic nerve has the same organization as the white matter of the brain, particularly when the constitution of glia and the organization of vasculature in the two structures are compared [133].

### 2.5. Energy Metabolism in Glial cells

The exact mechanisms leading to apoptosis in glaucoma are unclear. However, it is clear, other than retinal ganglion cells and their axons; the apoptosis also results in the destruction of supporting glial cells leading to a characteristic excavation of the ONH [189]. Hypoxic stress is observed to increase in the astrocytes in the ONH of glaucoma patients [190,191]. The presence of oxidative injury is also detected in astrocytes in the pre-laminar optic nerve head in human primary open-angle glaucoma [192]. It is also showed that activated astrocytes respond to increased IOP with protective factors; the astrocytes in human glaucomatous optic nerve heads exhibit increased expression of glutamate-cysteine ligase, the rate-limiting enzyme of synthesis of glutathione (GSH) [189,193]. The antioxidant properties of GSH protect the mitochondrial electron transport chain from oxidative damage.

Glial cells protect and support the retinal ganglion cell axons in passing from the eye to the brain. The larger vessels of the inner retinal vasculature lay in the innermost portion of the retina, close to the inner limiting membrane. Their walls are in close spatial relationship with glial cells, mainly astrocytes, which constrain the vessels to the inner retina and maintain their integrity [194,195]. As well in the optic nerve head, almost 50% of the cells comprise glia [196], and astrocytes form a major part of this population and form glial tubes in the prelaminar part of the optic nerve through, which bundles of axons run to enter the optic nerve [189,197]. They are pivotal for the maintenance of the appropriate cellular environment for RGC axons. The gap junctions that connect the astrocytes act as a syncytium to buffer changes in the extracellular environment of the axon [198].

The astrocyte-neuron lactate shuttle (ANLS) hypothesis is proposed by Pellerin and Magistretti et al. [199]. They provided evidence that uptake of glutamate by astrocytes results in the activation of the Na+/K+ ATPase, which triggers glucose uptake and its glycolytic processing [200]. Glucose is then metabolized to lactate, contributing to the activity-dependent fueling of the neuronal energy demands associated with synaptic transmission [200,201]. They hypothesized that astrocytic lactate production is calibrated by neuronal glutamate production, proposing that there is feedback between energy supply and neurotransmission demands. It is worth noting that this concept is controversial in the field.

While glutamate induced a robust metabolic response in astrocytes (decreased ATP levels and glucose uptake stimulation), GABA does not couple inhibitory neuronal activity with glucose utilization [202].

Whether RGCs utilized lactate from glia cells as fuel is unclear. There is evidence of widespread lactate dehydrogenase (LDH) activity throughout the retina. The activity is predominantly derived from LDH-B in the inner retina. It is demonstrated that the oxygen-regulated expression of LDH-B is opposite and complementary to that of LDH-A [197]. In both vascular and avascular retinal cells, the LDH-B gene was repressed after hypoxia and reactivated after oxygen reperfusion [203]. LDH-B is also potently expressed in RGCs. However, glucose is the preferred energy substrate of retinal neurons; it is possible that other energetic substrates like lactate can also be utilized by RGCs. This hypothesis, however, needs to be further clarified. Overall, the ANLS model does not adequately explain many key features of the metabolic relationships between neurons and glia [204,205].

## 3. Conclusions

The high energy demands of the retina render it vulnerable to energy insufficiency. The inner retina is particularly susceptible to impaired energy metabolism due to its optical function. The energy demands of the neurons are tightly coupled with their functional activity. Each compartment of RGC is located in different retinal layers, therefore, different extracellular environments and have specific energy requirements. The energy homeostasis of RGC is complex and dependent upon a delicately regulated system to meet regional metabolic demands. The energetics of RGC compartments and their support system is important for helping us better understand the pathogenesis of glaucoma and develop a potential treatment for this leading cause of blindness throughout the globe.
